# Regional Responses in Radiation-Induced Normal Tissue Damage

**DOI:** 10.3390/cancers13030367

**Published:** 2021-01-20

**Authors:** Daniëlle C. Voshart, Julia Wiedemann, Peter van Luijk, Lara Barazzuol

**Affiliations:** 1 Department of Radiation Oncology, University of Groningen, University Medical Center Groningen, 9700 RB Groningen, The Netherlands; d.c.voshart@umcg.nl (D.C.V.); j.wiedemann@umcg.nl (J.W.); 2 Department of Biomedical Sciences of Cells & Systems–Section Molecular Cell Biology, University of Groningen, University Medical Center Groningen, 9700 RB Groningen, The Netherlands

**Keywords:** radiotherapy, normal tissue, regional effects, side effects, brain, salivary gland, lung heart, pancreas, bladder

## Abstract

**Simple Summary:**

Side effects caused by the concomitant irradiation of normal tissue during radiotherapy for cancer treatment can negatively affect the patient’s quality of life and limit the radiation dose that can safely be administered to the tumor. Recently, considerable developments have been achieved in radiotherapy and imaging technologies, allowing the selective sparing of the regions within organs that contribute most to the development of these side effects. This review discusses regional variation in the response to radiation in several organs, including the brain, salivary glands, cardiopulmonary system, pancreas, and bladder. Regional responses are shown to originate from general principles, such as the localization of target cells or function. We conclude that regional responses are a general phenomenon that should be studied in other organs. This will facilitate further optimization of the use of modern radiotherapy technologies.

**Abstract:**

Normal tissue side effects remain a major concern in radiotherapy. The improved precision of radiation dose delivery of recent technological developments in radiotherapy has the potential to reduce the radiation dose to organ regions that contribute the most to the development of side effects. This review discusses the contribution of regional variation in radiation responses in several organs. In the brain, various regions were found to contribute to radiation-induced neurocognitive dysfunction. In the parotid gland, the region containing the major ducts was found to be critical in hyposalivation. The heart and lung were each found to exhibit regional responses while also mutually affecting each other’s response to radiation. Sub-structures critical for the development of side effects were identified in the pancreas and bladder. The presence of these regional responses is based on a non-uniform distribution of target cells or sub-structures critical for organ function. These characteristics are common to most organs in the body and we therefore hypothesize that regional responses in radiation-induced normal tissue damage may be a shared occurrence. Further investigations will offer new opportunities to reduce normal tissue side effects of radiotherapy using modern and high-precision technologies.

## 1. Introduction

Radiotherapy remains a mainstay in cancer treatment. Over 50% of cancer patients receive radiotherapy [1,2], accounting for over 3 million people every year in Europe [3]. Improvements in treatments for cancer have considerably increased the life expectancy of patients. However, this leaves more patients at risk of developing side effects from their treatment. These side effects impact the patient’s future quality of life. This frequently limits the radiation dose that can be administered to the tumor, potentially reducing local tumor control. As such, reducing side effects is crucial.

To achieve this, radiotherapy technology is continuously being developed to improve precision. Examples of such developments are transitions from early photon-based techniques, like 3D conformal radiotherapy, to more recent techniques such as intensity modulated radiotherapy, volumetric modulated arc therapy, and the use of image-guidance. In addition, the use of particle-based therapies is increasing. Due to their physical properties, particles often allow a large reduction in dose to the normal tissues. In contrast to photons, most of the energy of charged particles is released in the Bragg peak, which can be positioned to the target volume [4]. Each of these developments aims at improving the conformality of the dose distribution to the targeted volume, reducing dose to the surrounding normal tissue.

The increased precision of modern radiotherapy technologies can be used to spare radiosensitive tissues. The term radiosensitivity is broadly used in radiation oncology as the susceptibility to develop radiation-induced side effects [5,6]. Radiosensitivity can be used to describe the response of different cell types, tissues or organs. Until the middle of the 1990s, radiosensitivity within a tissue or organ was thought to intrinsically relate to the response and loss of specific cells. This target cell hypothesis was appropriately reviewed by Bentzen et al. [7]. Early biological responses are largely an outcome of the DNA damage response encompassing DNA repair pathways, cell cycle checkpoints, and ultimately cell death, and can be cell-type-specific. For example, fast proliferating tissues, such as those in the intestine and skin, can undergo apoptosis or mitotic catastrophe within days after irradiation, in contrast to nervous or glandular tissue characterized by a slower turnover [8]. Irradiation of these fast proliferating tissues can cause early side effects, which are temporary and usually manifest during or within weeks after the completion of radiotherapy treatment. Examples are skin rash, mucositis, nausea and diarrhea. Late side effects are often chronic, appearing months or years after treatment. The extent of late side effects generally depends on the organ and the substructure within the organ that has received radiation. Examples range from dry mouth, hormonal dysfunction, neurocognitive impairment, gastro-intestinal problems, metabolic disorders, cardiac failure, and infertility, to secondary cancers [2,7]. These late responses are generally more complex; they include multiple cell types and biological pathways involved in processes such as inflammation, fibrogenesis, and vascular damage [7]. The target cells for these late effects may be distributed non-uniformly, such as the Islets of Langerhans in the pancreas [9]. Similarly, local sub-structures, such as well-perfused regions of the lung, can be critical for organ function [10]. Therefore, the field of radiobiology has investigated the existence of target regions and structures within a tissue/organ that contribute most to the pathogenesis of late side effects. The identification of regional responses can improve the understanding of mechanisms leading to late side effects. This will allow the optimal use of the increased precision offered by modern radiotherapy technologies by using region- and substructure-based objectives for radiotherapy treatment planning to achieve better sparing of identified critical regions and structures.

A number of preclinical and clinical studies have tried to identify such regions/structures within tissues and organs. In this review, we discuss regional variation in radiation-induced normal tissue damage in several organs, addressing whether they play a significant role in their functional response. We discuss if these regional responses are rare or if their occurrence in organs can be considered a general phenomenon.

## 2. Data by Organ

### 2.1. The Brain

**Figure 1:**
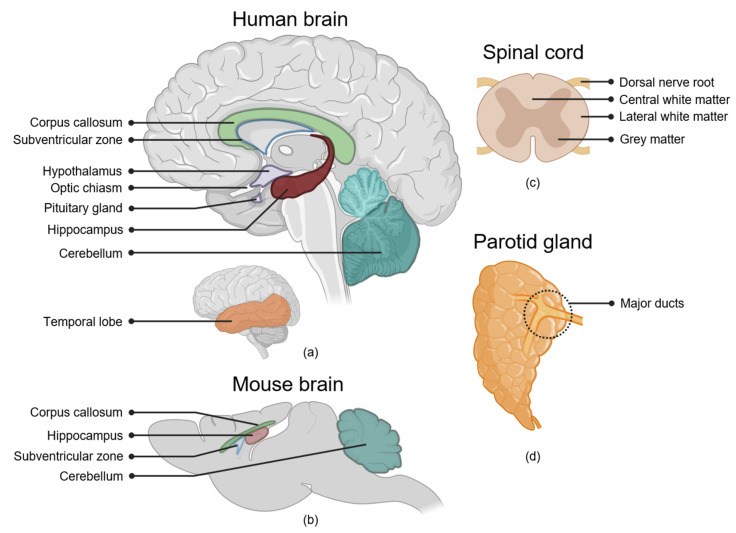
Organ regions associated with side effects of radiotherapy. (**a**) Locations of several brain regions showing regional responses to irradiation. Dose to the hippocampus, subventricular zone, corpus callosum, temporal lobe and cerebellum are associated with radiation-induced neurocognitive decline. The anterior cerebellum is indicated in a lighter color, the posterior cerebellum is indicated in a darker color. Irradiation of the hypothalamus and pituitary gland can lead to endocrine dysfunction. Irradiation of the optic chiasm and optic nerve—connecting to the anterior side of the optic chiasm—can result in radiation-induced optic neuropathy. (**b**) For comparison location of several of these regions in the mouse brain. (**c**) Location of different regions and structures in the spinal cord. Radiosensitivity of these regions differs between species and between the lumbar and cervical regions of the spinal cord. (**d**) Location of the major ducts, the putative stem cell-containing region, of the parotid gland.

The radiation-induced side effects of the brain include neurocognitive dysfunction, endocrine dysfunction, and neurosensory impairment [11,12,13]. Based on its composition of highly specialized interdependent sub-structures, regional responses have been hypothesized for brain function, and significant effort has been made to identify regions of the brain that might be particularly important for the development of radiation-induced side effects (Figure 1a,b). These regions are not mutually exclusive. Specifically, the white matter (WM) tracts are spread throughout the brain and are an important component of other regions described in this review, such as the cortex and cerebellum.

#### 2.1.1. Hippocampus

The hippocampus is a bilateral structure located in the brain temporal lobe. The hippocampus consists of several sub-structures, including the dentate gyrus (DG) containing the subgranular zone (SGZ). Together with the subventricular zone (SVZ) of the lateral ventricles, the SGZ is considered the primary region of neurogenesis in the adult mammalian brain [14]. The main cognitive functions of the hippocampus are learning, consolidation and retrieval of information. Additionally, hippocampal neurogenesis plays a key role in memory formation [14], as impairment of adult neurogenesis is thought to be linked to neurocognitive dysfunction like observed in Alzheimer’s disease [15,16].

Irradiation of the hippocampus leads to early loss of proliferating progenitor cells and immature neurons in the SGZ [17]. Whole-brain irradiation in both young and adult mice can lead to a persistent decline in neurogenesis [18,19,20]. In terms of cognitive function, focal irradiation of the hippocampus induced a decline in learning and (spatial) memory as measured by contextual fear conditioning in adult mice [21] and rats [22], and the Barnes maze test in adult mice [19]. In line with this, in adult mice, hippocampal sparing resulted in improved neurogenesis in the DG and rescued hippocampal-dependent spatial memory compared to whole-brain irradiation [23]. In these focal irradiation and sparing studies, some brain regions surrounding the hippocampus were also partly irradiated or spared respectively due to the collimator design, which could have partially affected the results. However, the strong hippocampal dependency of the behavioral tests used in these studies strengthens these findings.

In patients, a reduction in hippocampal volume has also been reported after radiotherapy [24,25,26] and a small study showed a decrease in neurogenesis in patients after treatment for central nervous system malignancies [27]. The radiation dose to the hippocampus has been associated with neurocognitive decline in several studies in pediatric patients. Acharya et al. found that increased hippocampal dose was associated with a stronger decline in delayed verbal recall scores in survivors of pediatric or adolescent low-grade glioma with a follow-up of 10 years [28]. In other studies, left hippocampal dose was specifically correlated to cognitive decline, indicating further regional variability [29,30,31]. This could be related to the mapping of specific brain functions in the two hemispheres of the brain. For example, language is primarily mapped to the left hemisphere [29]. Additionally, increased hippocampal dose has been associated with decreased motor speed and dexterity [32].

A correlation between dose to the hippocampus and verbal recall was found in adult patients in 6- to 18-month follow-up studies [33,34,35]. Based on this, several clinical trials in adult patients involving hippocampal sparing have started in recent years. In phase II and III clinical trials with 42 patients and 518 patients, respectively, hippocampal-sparing whole-brain radiotherapy (WBRT) showed better cognitive outcomes over time than WBRT without hippocampal sparing [36,37]. In conclusion, irradiation of the hippocampus contributes to cognitive decline in both pediatric and adult patients, likely due to neurogenesis and cognitive functions of the hippocampus.

#### 2.1.2. Subventricular Zone of the Lateral Ventricles

Similar to the SGZ, irradiation of the SVZ leads to apoptosis of neural progenitor cells [20,38]. However, in contrast to the SGZ region, proliferation in the SVZ recovers over time [20]. Proliferation in the SVZ is upregulated after injury, such as demyelination or stroke, and is thought to contribute to repair [39,40]. Focal irradiation of the SVZ in mice with 10 Gy only slightly impaired the ability of the SVZ to produce oligodendrocytes in response to a demyelinating lesion [41].

The risk of contrast-enhancing brain changes on magnetic resonance imaging (MRI) is increased in the periventricular region after irradiation [42,43]. These contrast-enhancing brain changes indicate increased blood–brain barrier permeability and can progress to radiation-induced brain lesions [43].

To what extent irradiation of the SVZ contributes to radiation-induced neurocognitive decline is still unclear. Studies in pediatric patients have not found a correlation between dose to the SVZ and cognitive decline [32,44]. In contrast, a retrospective study [45] and a prospective [46] study in adult patients found a correlation between SVZ dose and a decline in global cognition and delayed recall, respectively. Notably, glioblastoma stem cells have been hypothesized to originate in the SVZ [47,48] and increased dose to the SVZ has been associated with improved survival in glioblastoma patients in some, but not all, studies [48]. Altogether, although an association exists between SVZ irradiation and cognitive decline in adults, sparing the SVZ might impact cancer recurrence.

#### 2.1.3. Cerebral Cortex

The cortex is important for several higher-order cognitive processes. It consists of four major lobes: the frontal, occipital, parietal, and temporal lobes. Radiotherapy can cause dose-dependent cortical thinning, which is associated with cognitive decline [49]. Dose-dependent cortical thinning was found to occur in regions of the frontal, parietal, and temporal lobes, which are involved in higher-order cognitive functions, such as memory, attention, and executive function [50,51]. In contrast, no dose-dependent differences were found in primary cortical regions, such as the primary visual cortex belonging to the occipital lobe [50], suggesting that cortical thinning is a region-specific response.

Radiation dose to the temporal lobe has been associated with memory impairment in both pediatric [31,32,52,53,54] and adult patients [45,55]. This effect might be partly caused by the concomitant irradiation of the hippocampus, which is embedded in the temporal cortex. Dose to the orbitofrontal region in the frontal lobe has been associated with working memory decline [53]. In contrast, radiation dose to either the occipital or parietal regions has not been related to cognitive decline in pediatric patients [53,56].

#### 2.1.4. White Matter

Oligodendrocytes are glial cells whose main function is the insulation of axons with myelin sheets, thereby protecting the neurons and facilitating fast conduction of signals along the axons. In the brain, outside of the SVZ and the SGZ, the majority of cycling cells are oligodendrocyte precursor cells (OPCs). OPCs can undergo apoptosis within days after irradiation, followed by progressive demyelination months later [57]. Remyelination using grafted OPCs after irradiation has been shown to rescue memory and motor deficits in rats, indicating a contribution of WM injury to radiation-induced cognitive decline [58].

Radiation-induced WM changes can be monitored longitudinally using MRI-based imaging techniques, such as diffusion tensor imaging [59,60]. WM volume decreases progressively after radiation exposure. In pediatric patients, post-irradiation WM volume has been associated with declined motor and neurocognitive function, such as memory, attention, and learning deficits [61,62,63,64,65,66,67]. Radiation dose to the corpus callosum and left frontal WM was associated with cognitive decline in adults [45]. Additionally, pre-treatment WM injury was found to predict neurocognitive decline after radiotherapy treatment in a hippocampal-sparing study in adult patients. This implies a hippocampal-independent mechanism through which radiation-induced neurocognitive decline can occur [68].

Little is known about subregional differences in WM sensitivity. A small clinical study with 22 patients showed increased WM damage in the frontal lobe compared to the parietal lobe in pediatric medulloblastoma survivors who received the same radiation dose to both regions [69]. The frontal lobe is the last brain region to complete myelination during early adulthood, which could explain the higher sensitivity [70]. Regional differences in radiotherapy-induced WM changes in pediatric patients are frequently reported for the corpus callosum [71,72,73,74]. The effect of sparing of the genu, the anterior region of the corpus callosum, on cognitive function is currently being investigated in a clinical trial [75].

These effects might not all be due to direct irradiation of the WM. Beera et al. reported that focal irradiation of the WM region anterior commissure in young mice did not lead to a volume reduction in this region. In contrast, irradiation of two non-WM regions, the olfactory bulb and the SVZ, resulted in a volume reduction in the anterior commissure and other WM regions [76]. This indicates that WM volume differences might be partly due to off-target effects, instead of the radiosensitivity of the WM itself.

#### 2.1.5. Cerebellum

Posterior fossa tumors, located near the brainstem and the cerebellum, account for more than half of all pediatric brain tumors and 20% of all adult brain tumors. Progressive neurocognitive decline is frequently seen in these patients; however, the effect of radiation on the normal tissue of the cerebellum is not well understood [77]. The cerebellum might be more sensitive to irradiation-induced vascular damage. Irradiated mice and rats showed increased short-term blood–brain barrier permeability in the cerebellum compared to other regions [78,79] as well as decreased blood flow [79].

In addition to motor function, the cerebellum contributes to neurocognitive tasks such as language, processing speed, and working memory. Specifically, the posterior cerebellum is engaged during cognitive processing [80]. Radiation dose to the cerebellum has been associated with cognitive decline in pediatric and adult patients [45,81]. In pediatric patients, dose to the posterior cerebellum was also associated with lower scores in academic tests [81]. These studies suggest that the cerebellum can develop local radiation responses that may translate into functional deficits.

#### 2.1.6. Hypothalamus and Pituitary Gland

Irradiation of the hypothalamus and anterior pituitary gland can lead to endocrine dysfunctions, which are common in both pediatric and adult patients [13,82].

The hypothalamus and pituitary gland are localized close together and interact via the hypothalamic–pituitary–adrenal and –gonadal axis. Several studies showed primary function loss of the hypothalamus [83]. Growth hormone (GH) deficiency is the most prevalent endocrine dysfunction after hypothalamus-pituitary gland irradiation, occurring at relatively low doses [83]. Specifically, the GH regulation by the hypothalamus was found to be impaired in pediatric patients with GH deficiency, while the pituitary gland was still able to produce GH when stimulated, primarily showing damage to the hypothalamus [84]. In addition, in other clinical studies on radiation-induced GH deficiency, GH regulation was found to be intact [85].

#### 2.1.7. Optic Nerve and Optic Chiasm

The optic nerve—which is the structure connecting the retina to the brain—, and the optic chiasm—the region of the brain where the optic nerves cross, which is important for the transmission of the visual information from the optic nerve to the occipital lobe of the brain—are both seen as organs at risk in brain and head and neck tumors [11]. Irradiation of these structures can cause radiation-induced optic neuropathy. This constitutes loss of vision to one or both eyes after damage to the optic nerve or optic chiasm, respectively [11,86]. Together with the hypothalamus and the pituitary gland, the optic nerve and chiasm are sub-structures that are critical for non-cognitive functions. When aiming to reduce the side effects of brain irradiation, the brain is not only important for cognition, but also contains structures with functions and impact outside of this domain.

### 2.2. The Spinal Cord

The spinal cord is an organ at risk in the treatment of many tumors as well as metastases [87]. Depending on the affected nerves and the severity of their damage, spinal cord side effects can range from sensory deficits and pain to loss of motor function and paralysis. Regional variations in the response of the spinal cord to radiation have been investigated in various animal models.

#### White Matter

In rats and swine, neurological symptoms following irradiation of the cervical spinal cord were primarily related to WM necrosis, without clear damage to the grey matter (Figure 1c) [88,89,90,91]. In contrast, irradiation of the rat thoracolumbar spinal cord led to more damage in the dorsal nerve roots than to the WM. This suggests relative variations in sensitivity for the necrosis of the nerve roots and WM between the lumbar and cervical regions [92]. In addition to differences in response between white and grey matter, differences in radiosensitivity between the centrally- and laterally-located WM have been observed [89]. Although the exact mechanism has not been resolved, the lateral localization of OPCs has been suggested to be responsible for the higher radiosensitivity of the lateral edge as compared with the centrally-located WM. In this hypothesis, the repopulation of oligodendrocytes lost after irradiation is possible after irradiation of the central but not the lateral region [89]. Interestingly, although irradiating the lateral half of the rat spinal cord increased the tolerance dose compared to irradiating the whole cross-section [89], this volume-effect was not observed in a Yucatan swine model, where stereotactic irradiation of the lateral edge of the spinal cord did not lead to function sparing [90,91]. A possible explanation for the difference with the rat model may be found in the migration distance of OPCs. In rats this is limited to about 2 mm [93,94]. The size of the cross-section of the swine spinal cord varies from 8 to 11 mm, limiting the potential of repopulation by migrating OPCs [90]. Since the dimensions of the human spinal cord are similar, the role of regional responses and volume effects is expected to be limited in patients. However, due to the severity of spinal cord complications and consequent strict dose limits used, clinical data on spinal cord complications are and will remain rare. As such, conclusive studies on regional sensitivity of the human spinal cord will not likely be performed.

### 2.3. Salivary Glands

The use of radiotherapy in head and neck cancer patients inevitably comes with exposure to radiation of normal tissues such as the salivary glands. Dysfunction of salivary glands can lead to secondary complications such as xerostomia, loss of teeth, and problems with eating, speaking, and sleeping, which all strongly impact the patient’s quality of life [95,96,97]. The occurrence of xerostomia has been shown to relate to the radiation dose to the parotid glands, submandibular glands, and oral cavity, which contains a large number of minor glands [98,99,100,101].

#### Parotid Gland Major Ducts

Morphologically, the parotid gland consists of a ductal system that branches from the excretory duct toward their secretory units, the acini, both supported by stromal tissue. The acinar cells are capable of transporting water from the adjacent vasculature into the ductal system [102]. In rats, parotid gland irradiation can reduce saliva production without significant loss of acinar cells during the first 30 days after irradiation [103]. In this phase, loss of saliva production is related to impairment of the transport function by inducing acute membrane damage [104]. However, subsequently, tissue degeneration secondary to loss of proliferating cells and consequent disturbance of tissue homeostasis occur. In salivary glands, tissue homeostasis is supported by proliferation of acinar cells and ductal tissue-specific stem cells [105,106,107]. However, in rats, mainly ducts remained one year after irradiation [108,109]. In this setting, ductal cells are capable of generating acinar cells, contributing to the recovery of this tissue compartment [105,107].

The localization of these two cell populations may lead to regional variations in radiation response. Loss of saliva production was more pronounced after irradiation of the cranial half of the rat parotid gland compared to the caudal half [108]. This enhanced response corresponded with a global degeneration in salivary glandular tissue, including the shielded half [109]. Irradiation of even smaller parts of the gland revealed that this global degenerative response depends on the radiation dose to the major ducts [110] (Figure 1d). Within the same study, post-radiotherapy parotid gland saliva production in patients was found to be best predicted by the dose to the region near the dorsal edge of the mandible [110]. This region contains the major ducts of the gland, and tissue samples obtained from this region contained the highest number of tissue-specific stem/progenitor cells, leading to the highest regenerative capacity in an organoid culture system [110].

The current clinical approach to minimizing the risk of xerostomia is preservation of salivary function by minimizing the dose to the parotid glands in treatment planning. Several studies have indicated that post-radiotherapy parotid gland function and xerostomia are better predicted by dose to regions or spatial features of the dose distribution [110,111,112,113,114,115]. As mentioned above, a region adjacent to the dorsal edge of the mandible was identified as the best predictor of post-treatment function [110]. The influence of voxel dose in the parotid and submandibular glands on the occurrence of xerostomia was also evaluated in a larger retrospective study. This revealed that the apparent influential regions may vary between the ipsilateral and contralateral parotid glands [112]. Although in both glands the influential region includes the major ducts, the region appeared larger in the contralateral parotid gland. As indicated by the authors, such differences might have resulted from collinearity with dose elsewhere. However, in this cohort the extended region received a relatively low dose. A role for such low doses in the development of xerostomia is supported by the analysis of a larger patient cohort of 684 patients [116]. Together, these studies indicate that low radiation doses have a strong impact on the response of salivary glands. In line with this, in rats, low radiation doses combined with significant doses elsewhere were shown to strongly reduce post-irradiation function [117,118].

As frequently noted by authors of studies reporting regional or voxel-wise analyses, strong correlations between doses in different regions or even organs complicate the interpretation of the results. However, the combination with independent development and testing of the hypothesis in preclinical models provides confidence in the interpretations described above [119].

The saliva production and composition of the different glands varies with time of the day. For example, the parotid gland produces almost no saliva at night [120,121,122]. Consequently, the temporal manifestation of symptoms related to salivary gland damage and the specific glands involved may vary similarly [123,124]. To allow more specific assessment of the role of specific glands in xerostomia, a questionnaire was developed to measure day- and night-time xerostomia and sticky saliva [123]. However, an initial study using this questionnaire did not find differential roles of the different salivary glands in the development of xerostomia or sticky saliva [125]. A potential reason for this is that more detailed assessment of complications on multiple sub-scales inevitably leads to a reduced number of events per type of complication, thus reducing the power of the study to detect associations with dose to specific glands.

### 2.4. Cardiopulmonary System

In the treatment of thoracic tumors, coincidental dose to heart and lungs frequently leads to side effects such as pericarditis, cardiomyopathy, or myocardial fibrosis in the heart occurring months to years after radiotherapy, and early pneumonitis and late pulmonary fibrosis in the lungs. To address this, current practice is to minimize mean lung dose (MLD), mean heart dose (MHD), and/or volumes of these organs receiving a specified minimum dose [126]. However, these dose metrics do not contain information on the spatial dose distribution. Consequently, these approaches consider the lung as a single, paired and functionally uniform organ. Nevertheless, clinical studies have recognized that radiation-induced dyspnea occurs more frequently in patients with tumors in the lower lung lobe [127,128], suggesting that the use of spatial information might improve the prediction of radiation-induced side effects of the lung [126].

#### 2.4.1. Alveolar and Microvascular Dense Regions of the Lung

**Figure 2:**
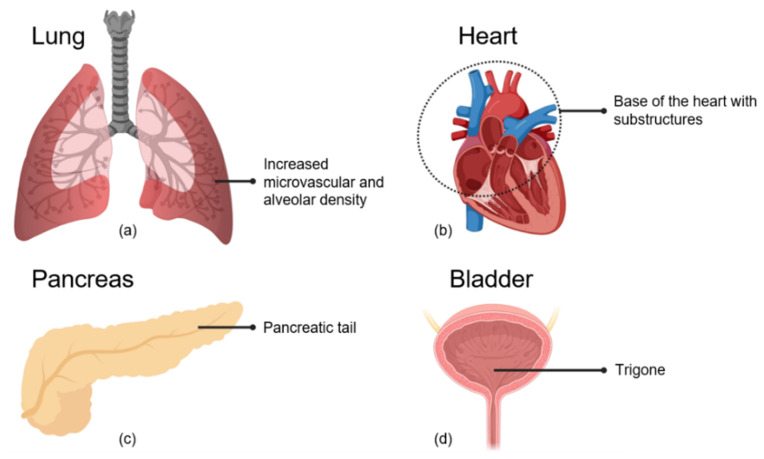
Organ regions associated with side effects of radiotherapy. (**a**) The lateral and basal regions of the lung show increased microvascular and alveolar density. Irradiation of these regions is associated with decreased survival and respiratory rate enhancement in preclinical studies. (**b**) Irradiation of structures in the base of the heart or of the major vessels connected to the base of the heart is associated with increased functional changes and mortality. (**c**) Irradiation of the pancreatic tail is associated with a higher risk of developing diabetes. (**d**) Irradiation of the trigone, located at the bladder base, is associated with late genitourinary side effects.

In several preclinical studies in mice and rats, more pronounced effects in terms of survival and respiratory rate enhancement were observed after irradiation of the basal parts of the lung compared to the apical parts [129,130,131,132]. A hypothesis explaining these differences was a non-uniform distribution of functional subunits within the lung [129,133]. In the lung, the alveoli are responsible for gas exchange and therefore essential for organ function. These are mainly located in the basal and lateral regions of the lungs [130] (Figure 2a). Using a mouse model, Travis et al. showed that these regional responses are related to the location of alveoli-rich regions [130]. In a rat model, more severe changes in function and histology also occurred after irradiation of the lateral parts of the lung compared to the mediastinal parts [134].

The effect of lung irradiation is not limited to the irradiated part, but also occurs in surrounding shielded tissue [131,132,135,136]. For instance, micronuclei formation in non-irradiated areas has been suggested to relate to the release of cytokines or reactive oxygen species (ROS) formation in irradiated areas [131,132].

In addition to the alveoli, the pulmonary vasculature was found to be a target for radiation-induced loss of function [135]. Damage to endothelial cells of small- and medium-sized vessels leads to disruption of the endothelial lining and to non-functional vasculature. Secondary to reduced vascular capacity in the irradiated region, vasculature in non-irradiated lung regions can be damaged due to enhanced pressure and overload [135]. Since small- and medium-sized vessels colocalize with the alveolar tissue [137] (Figure 2a), their respective roles in the development of the loss of pulmonary function cannot be clearly distinguished. However, most likely, both contribute to the radiation effects on lung function after irradiation of lateral parts of the lung, whereas mediastinal areas, containing more primary and secondary bronchi and bigger vessels, are more resistant.

A relation between the irradiation of different lung regions and the occurrence of radiation-induced lung injury was also reported in patients. Dose to patient-specific regions exhibiting a higher density before radiotherapy was predictive of post-treatment damage. These regions were mainly found in the base of the lung, likely indicating functionally important areas [138]. Interestingly, hypo-fractionated stereotactic radiotherapy of tumors close to the proximal bronchial tree (PBT), a defined area containing the main bronchi, is associated with a 3-fold increased risk of non-cancer death compared to patients with peripheral tumors. Although the endpoint is not specific for pulmonary side effects, this finding may suggest a role for the major bronchi or associated large pulmonary vessels in the development of severe toxicity when small volumes of lung are irradiated with high doses [139].

#### 2.4.2. Basal Region of the Heart

As the heart is an organ which consists of several different structures important for organ function, like valves and big vessels, it is likely that there are differences in the radiation response of these structures. Since precise irradiation of specific structures of the heart is challenging in small animal models, only few preclinical studies on the sensitivity of cardiac substructures are available [140]. A recent study by Ghita et al. showed that the basal parts of the heart are more sensitive to irradiation [141] (Figure 2b). Mean heart dose was not found to be a reliable predictor of functional changes after irradiation of cardiac sub-volumes in a mouse model. The observation of more pronounced effects after irradiation of the base of the heart indicated the presence of sensitive substructures in this part. These could potentially be related to the presence of the aortic and mitral valves, the pulmonary and coronary arteries, and the superior vena cava [141].

These findings are in line with several studies in patients reporting associations between dose to structures at the base of the heart and outcome. In studies in non-small cell lung cancer (NSCLC) patients, non-cancer mortality was associated with irradiation of upper regions of the heart, including big vessels (e.g., the vena cava and coronary arteries, AV-node, and sinus) and the right atrium [142,143,144]. Dose to the superior vena cava can especially be related to non-specific electrocardiogram abnormalities in NSCLC patients [145].

#### 2.4.3. Heart and Lung Interaction

Clinically, both lung and heart frequently receive a non-negligible radiation dose that can lead to radiation-induced side effects. In rats, an enhancement of radiation damage was found when irradiating both heart and lung [146]. In this preclinical model, damage and remodeling of pulmonary vasculature led to higher right ventricle (RV) systolic pressure and RV hypertrophy. In turn, these effects contributed to reduced left ventricle (LV) diastolic function [147]. In addition, irradiation of the heart can cause myocardial damage, reducing diastolic function. The consequential congestion in the pulmonary vasculature causes interstitial edema, parenchymal inflammation, and fibrosis in lung tissue [147]. Irradiation of both the heart and parts of the lung can thus directly and indirectly impair LV function via the aforementioned mechanisms, leading to aggravated cardiopulmonary dysfunction [147].

Clinical studies regarding the impact of heart irradiation on lung function had varying outcomes and conclusions. A large retrospective study including around 600 patients did not find an impact of heart irradiation on lung function [148], whereas another study with more than 200 patients reported a relation [149]. However, both studies tried to correlate heart dose with the occurrence of radiation pneumonitis, which is, according to the mentioned animal studies, not the only endpoint to be considered. Increased pulmonary artery pressure and a reduction in diastolic function should be considered as endpoints as well. However, these are currently not part of standard assessments of the side effects of radiotherapy.

### 2.5. The Pancreas

The pancreas has both endocrine and exocrine functions. Radiation-induced injury of the endocrine pancreas is known to increase the risk of diabetes mellitus [150,151].

The concentration of islets of Langerhans is higher in the pancreatic tail than in the rest of the pancreas [9] (Figure 2c). Retrospective studies have shown that irradiation of this region is associated with a higher risk of developing diabetes in both childhood cancer survivors [151,152] and adult patients [153].

### 2.6. The Bladder

Radiotherapy remains a mainstay in the management of cancers in the pelvic region, including cancers to the rectum, urinary bladder, uterus, ovary, and prostate. Bladder irradiation is associated with acute and late genitourinary (GU) side effects such as cystitis. This affects the quality of life of a significant portion of patients [154]. A review by Zuppone et al. [155] of the current status of research on radiation-induced bladder complications highlighted the lack of pre-clinical studies on the identification of critical sub-structures within the bladder. The investigations of possible bladder sub-regions predictive of late GU complications have been mostly based on a limited number of clinical studies and have found a spatial effect of the trigone region [155] (Figure 2d). In a recent study, the urethra and posterior regions above the trigone have also been identified as more predictive for urinary toxicity than the dose to the whole bladder [156].

#### Bladder Trigone

The trigone is a triangular region located at the bladder base just above the bladder neck. Its functions include preventing urine reflux and signaling the need for voiding [157]. A number of studies in prostate cancer patients have reported that mean dose or dose hotspots to the trigone are associated with late GU side effects [158,159,160,161]. Although the trigone region has been reported to influence the function of the bladder neck by causing obstruction that can prevent normal function [160], the underlying mechanism of this association is not yet clear.

## 3. Discussion

As the life expectancy of cancer patients increases, reducing radiotherapy-induced side effects is becoming more important to preserve post-treatment quality of life. Continuous improvements in radiotherapy technologies allow further reduction in radiation dose to normal tissues as well as specific sparing of regions and sub-structures of organs. As indicated by the reviewed literature, a considerable reduction in side effects may be achieved by selective sparing of specific regions within organs. In various organs regional responses have been identified. Examples include critical roles of the hippocampus in cognitive dysfunction, the ductal region of the parotid gland in hyposalivation, and the tail of the pancreas in diabetes in patients [36,110,151]. These regional responses are based on general principles like non-uniform distribution of target cells or the existence of sub-structures critical for function. Other organs that have not yet been studied in similar detail possess similar characteristics. This suggests that regional responses are a common phenomenon that can potentially offer opportunities to further optimize radiotherapy.

Several approaches to obtain insight into regional effects have been used and were described in the above organ-specific sections. Clinical studies benefit from directly investigating radiation-induced side effects and their impacts in patients. However, the multidisciplinary nature of oncological treatments creates challenges for specifying the contribution of radiotherapy to side effects. For example, recent studies using immunotherapy and radiotherapy have highlighted an increased risk of developing MRI-based imaging changes [162] and side effects [163]. This demonstrates that combined treatments may modify the response of normal tissues to radiation. In addition, most clinical studies are based on retrospective analyses. Regardless of whether these data were collected retrospectively or in the context of prospective studies performed for other purposes, testing hypotheses regarding the role of target regions and structures was not usually considered in the initial study design. General challenges encountered in such studies are the occurrence of confounding factors and correlations between doses to different regions. Both complicate identifying the factor responsible for the observed regional response. For instance, the observation that rectal bleeding is predominantly associated with dose to the anterior rectum wall in prostate cancer patients, reflecting the location of the prostate and the consistent inclusion of this part of the rectum in the target volume [164]. Similarly, parotid gland dose strongly correlates with dose to its sub-volume containing the putative tissue stem cells. To some extent, these limitations can be overcome by performing prospective studies with a design optimized for elucidating the role of confounding factors and/or reduce collinearity by randomizing patients between different treatment planning strategies [113,114]. However, patients need to be treated adequately. This poses limits on acceptable modifications to the radiotherapy treatment plan for investigating side effects. In addition, such studies usually still rely on associations and often lack proof for the hypothesized mechanisms leading to the regional variation. Hence, quality evidence for regional responses including their mechanistic basis can only be obtained by combining clinical and preclinical studies. The latter allow detailed investigation of mechanisms and the use of dose distributions optimized for hypothesis testing. For example, dose distributions used in studies of cardiopulmonary side effects differed strongly from clinical practice to allow critical testing of the role of heart and lung [134,146,147].

Nevertheless, preclinical studies are also subject to limitations that are not always recognized. Biological responses are often strongly species-dependent [165]. Examples of these include dose-limiting complications varying between radiation pneumonitis and pleural effusions depending on which mouse strain is used, as well as tolerance doses varying by a factor of two between mouse strains [166]. Similarly, radiation-induced alterations in macrophages have been shown to be mouse-strain-specific, indicating that choosing the right strain is critical for a meaningful clinical translation [167]. As such, investigating hypothesized mechanisms underlying regional responses observed in patients requires choosing an animal model in which this mechanism plays a role in the response to radiation. Although there are anatomical differences between rodents and humans in for example the brain (Figure 1a,b) and quantitative translation cannot be made, the function and overall cognitive domains are maintained between these species. Therefore, animal models can be used for hypothesis-generating and proof-of-concept studies. Translating results into clinical studies often requires an intermediate translational step to confirm the role of a mechanism in patients. This can be achieved in small clinical studies. For example, in a proof-of-concept study, cardiac MRI was used to directly assess changes in cardiopulmonary blood flow as a mechanism-specific endpoint [168]. In the brain, conventional MRI is used to track white and grey matter changes. The integrity of WM can further be studied using diffusion tensor imaging [60]. Functional MRI can be used to check the activation of cortical areas during specific tasks and, importantly, investigate the effect of radiotherapy on neural networks [169]. The brain is a highly complex organ with both intra- and interregional circuits. Damage to a component of these circuits can be compensated by another component or lead to function decline, and research into the effect of radiation on neural networks can help us to further understand regional responses. Being non-invasive and often providing mechanism-specific information, the use of imaging is attractive in this type of study. However, endpoints can sometimes also be made more mechanism-specific without the need for imaging. Xerostomia is usually registered as a general phenomenon. However, due to the parotid gland’s specific role in eating and its inactivity during the night, this is not an optimal endpoint when investigating strategies to reduce parotid gland-related side effects. Using a questionnaire distinguishing between day- and night-time complaints could help specify the affected glands [123,124].

A common challenge in clinical and preclinical work is that more detailed regional responses can only be investigated when technology is available. Clinically, association studies aiming at detecting regional variations require that the technology used can deliver radiation dose distributions with sufficient spatial variation to induce a detectable impact of regional responses on the clinical side effect. This implies that available clinical data usually lack information on the potential impact of the use of new, improved-precision radiotherapy when the latter becomes available. In this setting, the hypotheses generated in preclinical research can contribute to the optimal use of unique features of this new technology. The role of preclinical research for this purpose may increase with the recent availability of dedicated small animal irradiators in combination with the availability of histological information at the cellular level that can potentially be overlaid with the given radiation dose [170].

As indicated, regional responses may be a common phenomenon. Several organs would be of particular interest to study. The kidney consists of structurally separated tissues with several potential stem and progenitor cell niches [171]. Radiotherapy treatment in the kidney can result in late complications, such as nephropathy [172]. Studies including functional and imaging follow-up together with radiation dose distribution, like the RAPRASI study [173], are needed to clarify if any regional variation occurs in the radiation response that might be exploited to reduce side effects. Another interesting organ is the uterus. Irradiation of the uterus, specifically during childhood, increases the risk of infertility and adverse obstetrical outcomes later in life, such as miscarriage, neonatal death, and reduced birthweight [174,175,176]. Fulfilling its full function from conception to delivery, the uterus has to perform a large number of functions, each relying on one or more specific anatomical sub-structures. This might also lead to different regional responses to radiotherapy. The rectum and anal canal may be interesting candidates for further studies. Fecal incontinence correlates with the dose to the lower rectum and, more specifically, to the anal canal [177]. It has been hypothesized that this regional response may relate to the spatial distribution of the enteric nervous system [178,179]. Nevertheless, due to the paucity of preclinical studies on this subject, the pathophysiology and underlying biological mechanisms for such regional variation still need to be clarified [178].

## 4. Conclusions

This review discussed current pre-clinical and clinical evidence of regional responses in radiation-induced normal tissue damage in a number of organs at risk for development of side effects after radiotherapy. These regional responses were shown to originate from rather general principles, which are present in most organs. Taken together, we conclude that regional responses are a general phenomenon that needs to be studied in other organs to facilitate further optimization of the use of modern technology in radiotherapy.

## References

[1] Delaney G., Jacob S., Featherstone C., Barton M. (2005). The Role of Radiotherapy in Cancer Treatment. Cancer.

[2] De Ruysscher D., Niedermann G., Burnet N.G., Siva S., Lee A.W.M., Hegi-Johnson F. (2019). Radiotherapy Toxicity. Nat. Rev. Dis. Prim..

[3] Borras J.M., Lievens Y., Barton M., Corral J., Ferlay J., Bray F., Grau C. (2016). How Many New Cancer Patients in Europe Will Require Radiotherapy by 2025? An ESTRO-HERO Analysis. Radiother. Oncol..

[4] Loeffler J.S., Durante M. (2013). Charged Particle Therapy—Optimization, Challenges and Future Directions. Nat. Rev. Clin. Oncol..

[5] Seibold P., Auvinen A., Averbeck D., Bourguignon M., Hartikainen J.M., Hoeschen C., Laurent O., Noël G., Sabatier L., Salomaa S. (2020). Clinical and Epidemiological Observations on Individual Radiation Sensitivity and Susceptibility. Int. J. Radiat. Biol..

[6] Britel M., Bourguignon M., Foray N. (2018). The Use of the Term ‘Radiosensitivity’ through History of Radiation: From Clarity to Confusion. Int. J. Radiat. Biol..

[7] Bentzen S.M. (2006). Preventing or Reducing Late Side Effects of Radiation Therapy: Radiobiology Meets Molecular Pathology. Nat. Rev. Cancer.

[8] Dörr W., Joiner M.C., van der Kogel A.J. (2009). Pathogenesis of Normal-Tissue Side-Effects. Basic Clinical Radiobiology.

[9] Wittingen J., Frey C.F. (1974). Islet Concentration in the Head, Body, Tail and Uncinate Process of the Pancreas. Ann. Surg..

[10] Kocak Z., Borst G.R., Zeng J., Zhou S., Hollis D.R., Zhang J., Evans E.S., Folz R.J., Wong T., Kahn D. (2007). Prospective Assessment of Dosimetric/Physiologic-Based Models for Predicting Radiation Pneumonitis. Int. J. Radiat. Oncol. Biol. Phys..

[11] Mayo C., Martel M.K., Marks L.B., Flickinger J., Nam J., Kirkpatrick J. (2010). Radiation Dose–Volume Effects of Optic Nerves and Chiasm. Int. J. Radiat. Oncol. Biol. Phys..

[12] Lawrence Y.R., Li X.A., el Naqa I., Hahn C.A., Marks L.B., Merchant T.E., Dicker A.P. (2010). Radiation Dose–Volume Effects in the Brain. Int. J. Radiat. Oncol. Biol. Phys..

[13] Chemaitilly W., Li Z., Huang S., Ness K.K., Clark K.L., Green D.M., Barnes N., Armstrong G.T., Krasin M.J., Srivastava D.K. (2015). Anterior Hypopituitarism in Adult Survivors of Childhood Cancers Treated with Cranial Radiotherapy: A Report from the St Jude Lifetime Cohort Study. J. Clin. Oncol..

[14] Gonçalves J.T., Schafer S.T., Gage F.H. (2016). Adult Neurogenesis in the Hippocampus: From Stem Cells to Behavior. Cell.

[15] Tobin M.K., Musaraca K., Disouky A., Shetti A., Bheri A., Honer W.G., Kim N., Dawe R.J., Bennett D.A., Arfanakis K. (2019). Human Hippocampal Neurogenesis Persists in Aged Adults and Alzheimer’s Disease Patients. Cell Stem Cell.

[16] Moreno-Jiménez E.P., Flor-García M., Terreros-Roncal J., Rábano A., Cafini F., Pallas-Bazarra N., Ávila J., Llorens-Martín M. (2019). Adult Hippocampal Neurogenesis Is Abundant in Neurologically Healthy Subjects and Drops Sharply in Patients with Alzheimer’s Disease. Nat. Med..

[17] Fike J.R., Rola R., Limoli C.L. (2007). Radiation Response of Neural Precursor Cells. Neurosurg. Clin. N. Am..

[18] Mizumatsu S., Monje M.L., Morhardt D.R., Rola R., Palmer T.D., Fike J.R. (2003). Extreme Sensitivity of Adult Neurogenesis to Low Doses of X-Irradiation. Cancer Res..

[19] Raber J., Rola R., LeFevour A., Morhardt D., Curley J., Mizumatsu S., VandenBerg S.R., Fike J.R. (2004). Radiation-Induced Cognitive Impairments Are Associated with Changes in Indicators of Hippocampal Neurogenesis. Radiat. Res..

[20] Hellström N.A.K., Björk-Eriksson T., Blomgren K., Kuhn H.G. (2009). Differential Recovery of Neural Stem Cells in the Subventricular Zone and Dentate Gyrus After Ionizing Radiation. Stem Cells.

[21] Saxe M.D., Battaglia F., Wang J.-W., Malleret G., David D.J., Monckton J.E., Garcia A.D.R., Sofroniew M.V., Kandel E.R., Santarelli L. (2006). Ablation of Hippocampal Neurogenesis Impairs Contextual Fear Conditioning and Synaptic Plasticity in the Dentate Gyrus. Proc. Natl. Acad. Sci. USA.

[22] Hernández-Rabaza V., Llorens-Martín M., Velázquez-Sánchez C., Ferragud A., Arcusa A., Gumus H.G., Gómez-Pinedo U., Pérez-Villalba A., Roselló J., Trejo J.L. (2009). Inhibition of Adult Hippocampal Neurogenesis Disrupts Contextual Learning but Spares Spatial Working Memory, Long-Term Conditional Rule Retention and Spatial Reversal. Neuroscience.

[23] Tomé W.A., Gökhan Ş., Brodin N.P., Gulinello M.E., Heard J., Mehler M.F., Guha C. (2015). A Mouse Model Replicating Hippocampal Sparing Cranial Irradiation in Humans: A Tool for Identifying New Strategies to Limit Neurocognitive Decline. Sci. Rep..

[24] Takeshita Y., Watanabe K., Kakeda S., Hamamura T., Sugimoto K., Masaki H., Ueda I., Igata N., Ohguri T., Korogi Y. (2020). Early Volume Reduction of the Hippocampus after Whole-Brain Radiation Therapy: An Automated Brain Structure Segmentation Study. Jpn. J. Radiol..

[25] Seibert T.M., Karunamuni R., Bartsch H., Kaifi S., Krishnan A.P., Dalia Y., Burkeen J., Murzin V., Moiseenko V., Kuperman J. (2017). Radiation Dose–Dependent Hippocampal Atrophy Detected with Longitudinal Volumetric Magnetic Resonance Imaging. Int. J. Radiat. Oncol. Biol. Phys..

[26] Nieman B.J., de Guzman A.E., Gazdzinski L.M., Lerch J.P., Chakravarty M.M., Pipitone J., Strother D., Fryer C., Bouffet E., Laughlin S. (2015). White and Gray Matter Abnormalities After Cranial Radiation in Children and Mice. Int. J. Radiat. Oncol. Biol. Phys..

[27] Monje M.L., Vogel H., Masek M., Ligon K.L., Fisher P.G., Palmer T.D. (2007). Impaired Human Hippocampal Neurogenesis after Treatment for Central Nervous System Malignancies. Ann. Neurol..

[28] Acharya S., Wu S., Ashford J.M., Tinkle C.L., Lucas J.T., Qaddoumi I., Gajjar A., Krasin M.J., Conklin H.M., Merchant T.E. (2019). Association between Hippocampal Dose and Memory in Survivors of Childhood or Adolescent Low-Grade Glioma: A 10-Year Neurocognitive Longitudinal Study. Neuro-Oncology.

[29] Zureick A.H., Evans C.L., Niemierko A., Grieco J.A., Nichols A.J., Fullerton B.C., Hess C.B., Goebel C.P., Gallotto S.L., Weyman E.A. (2018). Left Hippocampal Dosimetry Correlates with Visual and Verbal Memory Outcomes in Survivors of Pediatric Brain Tumors. Cancer.

[30] Goda J.S., Dutta D., Krishna U., Goswami S., Kothavade V., Kannan S., Maitre M., Bano N., Gupta T., Jalali R. (2020). Hippocampal Radiotherapy Dose Constraints for Predicting Long-Term Neurocognitive Outcomes: Mature Data from a Prospective Trial in Young Patients with Brain Tumors. Neuro-Oncology.

[31] Greenberger B.A., Pulsifer M.B., Ebb D.H., MacDonald S.M., Jones R.M., Butler W.E., Huang M.S., Marcus K.J., Oberg J.A., Tarbell N.J. (2014). Clinical Outcomes and Late Endocrine, Neurocognitive, and Visual Profiles of Proton Radiation for Pediatric Low-Grade Gliomas. Int. J. Radiat. Oncol. Biol. Phys..

[32] Redmond K.J., Mahone E.M., Terezakis S., Ishaq O., Ford E., McNutt T., Kleinberg L., Cohen K.J., Wharam M., Horska A. (2013). Association between Radiation Dose to Neuronal Progenitor Cell Niches and Temporal Lobes and Performance on Neuropsychological Testing in Children: A Prospective Study. Neuro-Oncology.

[33] Gondi V., Hermann B.P., Mehta M.P., Tomé W.A. (2012). Hippocampal Dosimetry Predicts Neurocognitive Function Impairment After Fractionated Stereotactic Radiotherapy for Benign or Low-Grade Adult Brain Tumors. Int. J. Radiat. Oncol. Biol. Phys..

[34] Ma T.M., Grimm J., McIntyre R., Anderson-Keightly H., Kleinberg L.R., Hales R.K., Moore J., Vannorsdall T., Redmond K.J. (2017). A Prospective Evaluation of Hippocampal Radiation Dose Volume Effects and Memory Deficits Following Cranial Irradiation. Radiother. Oncol..

[35] Okoukoni C., McTyre E.R., Ayala Peacock D.N., Peiffer A.M., Strowd R., Cramer C., Hinson W.H., Rapp S., Metheny-Barlow L., Shaw E.G. (2017). Hippocampal Dose Volume Histogram Predicts Hopkins Verbal Learning Test Scores after Brain Irradiation. Adv. Radiat. Oncol..

[36] Brown P.D., Gondi V., Pugh S., Tome W.A., Wefel J.S., Armstrong T.S., Bovi J.A., Robinson C., Konski A., Khuntia D. (2020). Hippocampal Avoidance During Whole-Brain Radiotherapy Plus Memantine for Patients with Brain Metastases: Phase III Trial NRG Oncology CC001. J. Clin. Oncol..

[37] Gondi V., Pugh S.L., Tome W.A., Caine C., Corn B., Kanner A., Rowley H., Kundapur V., DeNittis A., Greenspoon J.N. (2014). Preservation of Memory with Conformal Avoidance of the Hippocampal Neural Stem-Cell Compartment During Whole-Brain Radiotherapy for Brain Metastases (RTOG 0933): A Phase II Multi-Institutional Trial. J. Clin. Oncol..

[38] Barazzuol L., Ju L., Jeggo P.A. (2017). A Coordinated DNA Damage Response Promotes Adult Quiescent Neural Stem Cell Activation. PLoS Biol..

[39] Nait-Oumesmar B., Picard-Riéra N., Kerninon C., Baron-Van Evercooren A. (2008). The Role of SVZ-Derived Neural Precursors in Demyelinating Diseases: From Animal Models to Multiple Sclerosis. J. Neurol. Sci..

[40] Lindvall O., Kokaia Z. (2015). Neurogenesis Following Stroke Affecting the Adult Brain. Cold Spring Harb. Perspect. Biol..

[41] Capilla-Gonzalez V., Guerrero-Cazares H., Bonsu J.M., Gonzalez-Perez O., Achanta P., Wong J., Garcia-Verdugo J.M., Quiñones-Hinojosa A. (2014). The Subventricular Zone Is Able to Respond to a Demyelinating Lesion After Localized Radiation. Stem Cells.

[42] van West S.E., de Bruin H.G., van de Langerijt B., Swaak-Kragten A.T., van den Bent M.J., Taal W. (2016). Incidence of Pseudoprogression in Low-Grade Gliomas Treated with Radiotherapy. Neuro-Oncology.

[43] Bahn E., Bauer J., Harrabi S., Herfarth K., Debus J., Alber M. (2020). Late Contrast Enhancing Brain Lesions in Proton-Treated Patients with Low-Grade Glioma: Clinical Evidence for Increased Periventricular Sensitivity and Variable RBE. Int. J. Radiat. Oncol. Biol. Phys..

[44] Khatua S., Dhall G., O’Neil S., Jubran R., Villablanca J.G., Marachelian A., Nastia A., Lavey R., Olch A.J., Gonzalez I. (2010). Treatment of Primary CNS Germinomatous Germ Cell Tumors with Chemotherapy Prior to Reduced Dose Whole Ventricular and Local Boost Irradiation. Pediatr. Blood Cancer.

[45] Peiffer A.M., Leyrer C.M., Greene-Schloesser D.M., Shing E., Kearns W.T., Hinson W.H., Tatter S.B., Ip E.H., Rapp S.R., Robbins M.E. (2013). Neuroanatomical Target Theory as a Predictive Model for Radiation-Induced Cognitive Decline. Neurology.

[46] Gui C., Vannorsdall T.D., Kleinberg L.R., Assadi R., Moore J.A., Hu C., Quiñones-Hinojosa A., Redmond K.J. (2020). A Prospective Cohort Study of Neural Progenitor Cell-Sparing Radiation Therapy Plus Temozolomide for Newly Diagnosed Patients with Glioblastoma. Neurosurgery.

[47] Lee J.H., Lee J.E., Kahng J.Y., Kim S.H., Park J.S., Yoon S.J., Um J.-Y., Kim W.K., Lee J.-K., Park J. (2018). Human Glioblastoma Arises from Subventricular Zone Cells with Low-Level Driver Mutations. Nature.

[48] Altmann C., Keller S., Schmidt M.H.H. (2019). The Role of SVZ Stem Cells in Glioblastoma. Cancers.

[49] Nagtegaal S.H.J., David S., van der Boog A.T.J., Leemans A., Verhoeff J.J.C. (2019). Changes in Cortical Thickness and Volume after Cranial Radiation Treatment: A Systematic Review. Radiother. Oncol..

[50] Seibert T.M., Karunamuni R., Kaifi S., Burkeen J., Connor M., Krishnan A.P., White N.S., Farid N., Bartsch H., Murzin V. (2017). Cerebral Cortex Regions Selectively Vulnerable to Radiation Dose-Dependent Atrophy. Int. J. Radiat. Oncol. Biol. Phys..

[51] Nagtegaal S.H.J., David S., Snijders T.J., Philippens M.E.P., Leemans A., Verhoeff J.J.C. (2020). Effect of Radiation Therapy on Cerebral Cortical Thickness in Glioma Patients: Treatment-Induced Thinning of the Healthy Cortex. Neuro-Oncol. Adv..

[52] Merchant T.E., Kiehna E.N., Li C., Xiong X., Mulhern R.K. (2005). Radiation Dosimetry Predicts IQ after Conformal Radiation Therapy in Pediatric Patients with Localized Ependymoma. Int. J. Radiat. Oncol. Biol. Phys..

[53] Doger de Speville E., Robert C., Perez-Guevara M., Grigis A., Bolle S., Pinaud C., Dufour C., Beaudré A., Kieffer V., Longaud A. (2017). Relationships between Regional Radiation Doses and Cognitive Decline in Children Treated with Cranio-Spinal Irradiation for Posterior Fossa Tumors. Front. Oncol..

[54] Jalali R., Mallick I., Dutta D., Goswami S., Gupta T., Munshi A., Deshpande D., Sarin R. (2010). Factors Influencing Neurocognitive Outcomes in Young Patients with Benign and Low-Grade Brain Tumors Treated with Stereotactic Conformal Radiotherapy. Int. J. Radiat. Oncol. Biol. Phys..

[55] Hsiao K.-Y., Yeh S.-A., Chang C.-C., Tsai P.-C., Wu J.-M., Gau J.-S. (2010). Cognitive Function Before and After Intensity-Modulated Radiation Therapy in Patients with Nasopharyngeal Carcinoma: A Prospective Study. Int. J. Radiat. Oncol. Biol. Phys..

[56] Armstrong G.T., Jain N., Liu W., Merchant T.E., Stovall M., Srivastava D.K., Gurney J.G., Packer R.J., Robison L.L., Krull K.R. (2010). Region-Specific Radiotherapy and Neuropsychological Outcomes in Adult Survivors of Childhood CNS Malignancies. Neuro-Oncology.

[57] Panagiotakos G., Alshamy G., Chan B., Abrams R., Greenberg E., Saxena A., Bradbury M., Edgar M., Gutin P., Tabar V. (2007). Long-Term Impact of Radiation on the Stem Cell and Oligodendrocyte Precursors in the Brain. PLoS ONE.

[58] Piao J., Major T., Auyeung G., Policarpio E., Menon J., Droms L., Gutin P., Uryu K., Tchieu J., Soulet D. (2015). Human Embryonic Stem Cell-Derived Oligodendrocyte Progenitors Remyelinate the Brain and Rescue Behavioral Deficits Following Radiation. Cell Stem Cell.

[59] Connor M., Karunamuni R., McDonald C., White N., Pettersson N., Moiseenko V., Seibert T., Marshall D., Cervino L., Bartsch H. (2016). Dose-Dependent White Matter Damage after Brain Radiotherapy. Radiother. Oncol..

[60] Ajithkumar T., Price S., Horan G., Burke A., Jefferies S. (2017). Prevention of Radiotherapy-Induced Neurocognitive Dysfunction in Survivors of Paediatric Brain Tumours: The Potential Role of Modern Imaging and Radiotherapy Techniques. Lancet Oncol..

[61] Jacola L.M., Ashford J.M., Reddick W.E., Glass J.O., Ogg R.J., Merchant T.E., Conklin H.M. (2014). The Relationship between Working Memory and Cerebral White Matter Volume in Survivors of Childhood Brain Tumors Treated with Conformal Radiation Therapy. J. Neurooncol..

[62] Reddick W.E., Taghipour D.J., Glass J.O., Ashford J., Xiong X., Wu S., Bonner M., Khan R.B., Conklin H.M. (2014). Prognostic Factors That Increase the Risk for Reduced White Matter Volumes and Deficits in Attention and Learning for Survivors of Childhood Cancers. Pediatr. Blood Cancer.

[63] Partanen M., Bouffet E., Laughlin S., Strother D., Hukin J., Skocic J., Szulc-Lerch K., Mabbott D.J. (2018). Early Changes in White Matter Predict Intellectual Outcome in Children Treated for Posterior Fossa Tumors. Neuroimage Clin..

[64] Carey M.E., Haut M.W., Reminger S.L., Hutter J.J., Theilmann R., Kaemingk K.L. (2008). Reduced Frontal White Matter Volume in Long-Term Childhood Leukemia Survivors: A Voxel-Based Morphometry Study. Am. J. Neuroradiol..

[65] Rueckriegel S.M., Bruhn H., Thomale U.W., Hernáiz Driever P. (2015). Cerebral White Matter Fractional Anisotropy and Tract Volume as Measured by MR Imaging Are Associated with Impaired Cognitive and Motor Function in Pediatric Posterior Fossa Tumor Survivors. Pediatr. Blood Cancer.

[66] Mulhern R.K., Palmer S.L., Reddick W.E., Glass J.O., Kun L.E., Taylor J., Langston J., Gajjar A. (2001). Risks of Young Age for Selected Neurocognitive Deficits in Medulloblastoma Are Associated with White Matter Loss. J. Clin. Oncol..

[67] Edelmann M.N., Krull K.R., Liu W., Glass J.O., Ji Q., Ogg R.J., Sabin N.D., Srivastava D.K., Robison L.L., Hudson M.M. (2014). Diffusion Tensor Imaging and Neurocognition in Survivors of Childhood Acute Lymphoblastic Leukaemia. Brain.

[68] Bovi J.A., Pugh S.L., Sabsevitz D., Robinson C.G., Paulson E., Mehta M.P., Gondi V., Kundapur V., Shahin M.S., Chao S.T. (2019). Pretreatment Volume of MRI-Determined White Matter Injury Predicts Neurocognitive Decline After Hippocampal Avoidant Whole-Brain Radiation Therapy for Brain Metastases: Secondary Analysis of NRG Oncology Radiation Therapy Oncology Group 0933. Adv. Radiat. Oncol..

[69] Qiu D., Kwong D.L.W., Chan G.C.F., Leung L.H.T., Khong P.-L. (2007). Diffusion Tensor Magnetic Resonance Imaging Finding of Discrepant Fractional Anisotropy between the Frontal and Parietal Lobes after Whole-Brain Irradiation in Childhood Medulloblastoma Survivors: Reflection of Regional White Matter Radiosensitivity?. Int. J. Radiat. Oncol. Biol. Phys..

[70] Schneider J.F.L., Il’yasov K.A., Hennig J., Martin E. (2004). Fast Quantitative Diffusion-Tensor Imaging of Cerebral White Matter from the Neonatal Period to Adolescence. Neuroradiology.

[71] Redmond K.J., Hildreth M., Sair H.I., Terezakis S., McNutt T., Kleinberg L., Cohen K.J., Wharam M., Horska A., Mahone E.M. (2018). Association of Neuronal Injury in the Genu and Body of Corpus Callosum After Cranial Irradiation in Children with Impaired Cognitive Control: A Prospective Study. Int. J. Radiat. Oncol. Biol. Phys..

[72] Rashid A., Ram A.N., Kates W.R., Redmond K.J., Wharam M., Mark Mahone E., Horska A., Terezakis S. (2017). A Prospective Study of Corpus Callosum Regional Volumes and Neurocognitive Outcomes Following Cranial Radiation for Pediatric Brain Tumors. Child’s Nerv. Syst..

[73] Palmer S.L., Reddick W.E., Glass J.O., Gajjar A., Goloubeva O., Mulhern R.K. (2002). Decline in Corpus Callosum Volume among Pediatric Patients with Medulloblastoma: Longitudinal MR Imaging Study. Am. J. Neuroradiol..

[74] Makola M., Douglas Ris M., Mahone E.M., Yeates K.O., Cecil K.M. (2017). Long-Term Effects of Radiation Therapy on White Matter of the Corpus Callosum: A Diffusion Tensor Imaging Study in Children. Pediatr. Radiol..

[75] Redmond K. Neurocognitive Functioning with Genu-Sparing Whole Brain Radiation Therapy for Brain Metastases. https://clinicaltrials.gov/ct2/show/NCT03223922.

[76] Beera K.G., Li Y.-Q., Dazai J., Stewart J., Egan S., Ahmed M., Wong C.S., Jaffray D.A., Nieman B.J. (2018). Altered Brain Morphology after Focal Radiation Reveals Impact of Off-Target Effects: Implications for White Matter Development and Neurogenesis. Neuro-Oncology.

[77] Cantelmi D., Schweizer T.A., Cusimano M.D. (2008). Role of the Cerebellum in the Neurocognitive Sequelae of Treatment of Tumours of the Posterior Fossa: An Update. Lancet Oncol..

[78] Sándor N., Walter F.R., Bocsik A., Sántha P., Schilling-Tóth B., Léner V., Varga Z., Kahán Z., Deli M.A., Sáfrány G. (2014). Low Dose Cranial Irradiation-Induced Cerebrovascular Damage Is Reversible in Mice. PLoS ONE.

[79] Zhou K., Boström M., Ek C.J., Li T., Xie C., Xu Y., Sun Y., Blomgren K., Zhu C. (2017). Radiation Induces Progenitor Cell Death, Microglia Activation, and Blood-Brain Barrier Damage in the Juvenile Rat Cerebellum. Sci. Rep..

[80] Moore D.M., D’Mello A.M., McGrath L.M., Stoodley C.J. (2017). The Developmental Relationship between Specific Cognitive Domains and Grey Matter in the Cerebellum. Dev. Cogn. Neurosci..

[81] Merchant T.E., Sharma S., Xiong X., Wu S., Conklin H. (2014). Effect of Cerebellum Radiation Dosimetry on Cognitive Outcomes in Children with Infratentorial Ependymoma. Int. J. Radiat. Oncol. Biol. Phys..

[82] Crowne E., Gleeson H., Benghiat H., Sanghera P., Toogood A. (2015). Effect of Cancer Treatment on Hypothalamic–Pituitary Function. Lancet Diabetes Endocrinol..

[83] Rose S.R., Horne V.E., Howell J., Lawson S.A., Rutter M.M., Trotman G.E., Corathers S.D. (2016). Late Endocrine Effects of Childhood Cancer. Nat. Rev. Endocrinol..

[84] Schmiegelow M., Lassen S., Poulsen H.S., Feldt-Rasmussen U., Schmiegelow K., Hertz H., Müller J. (2000). Growth Hormone Response to a Growth Hormone-Releasing Hormone Stimulation Test in a Population-Based Study Following Cranial Irradiation of Childhood Brain Tumors. Horm. Res. Paediatr..

[85] Darzy K.H., Pezzoli S.S., Thorner M.O., Shalet S.M. (2005). The Dynamics of Growth Hormone (GH) Secretion in Adult Cancer Survivors with Severe GH Deficiency Acquired after Brain Irradiation in Childhood for Nonpituitary Brain Tumors: Evidence for Preserved Pulsatility and Diurnal Variation with Increased Secretor. J. Clin. Endocrinol. Metab..

[86] Kountouri M., Pica A., Walser M., Albertini F., Bolsi A., Kliebsch U., Bachtiary B., Combescure C., Lomax A.J., Schneider R. (2020). Radiation-Induced Optic Neuropathy after Pencil Beam Scanning Proton Therapy for Skull-Base and Head and Neck Tumours. Br. J. Radiol..

[87] Bhattacharya I.S., Hoskin P.J. (2015). Stereotactic Body Radiotherapy for Spinal and Bone Metastases. Clin. Oncol..

[88] Van der Kogel A.J. (1979). Late Effects of Radiation on the Spinal Cord; Dose-Effect Relationships and Pathogenesis. Ph.D. Thesis.

[89] Bijl H.P., van Luijk P., Coppes R.P., Schippers J.M., Konings A.W.T., van der Kogel A.J. (2005). Regional Differences in Radiosensitivity across the Rat Cervical Spinal Cord. Int. J. Radiat. Oncol. Biol. Phys..

[90] Medin P.M., Foster R.D., van der Kogel A.J., Sayre J.W., McBride W.H., Solberg T.D. (2011). Spinal Cord Tolerance to Single-Fraction Partial-Volume Irradiation: A Swine Model. Int. J. Radiat. Oncol. Biol. Phys..

[91] Medin P.M., Foster R.D., van der Kogel A.J., Sayre J.W., McBride W.H., Solberg T.D. (2013). Spinal Cord Tolerance to Single-Session Uniform Irradiation in Pigs: Implications for a Dose-Volume Effect. Radiother. Oncol..

[92] Philippens M.E.P., Pop L.A.M., Visser A.G., van der Kogel A.J. (2007). Dose-Volume Effects in Rat Thoracolumbar Spinal Cord: The Effects of Nonuniform Dose Distribution. Int. J. Radiat. Oncol. Biol. Phys..

[93] Franklin R.J.M., Gilson J.M., Blakemore W.F. (1997). Local Recruitment of Remyelinating Cells in the Repair of Demyelination in the Central Nervous System. J. Neurosci. Res..

[94] van Luijk P., Bijl H.P., Konings A.W.T., van der Kogel A.J., Schippers J.M. (2005). Data on Dose-Volume Effects in the Rat Spinal Cord Do Not Support Existing NTCP Models. Int. J. Radiat. Oncol. Biol. Phys..

[95] Vissink A., Jansma J., Spijkervet F.K.L., Burlage F.R., Coppes R.P. (2003). Oral Sequelae of Head and Neck Radiotherapy. Crit. Rev. Oral Biol. Med..

[96] Langendijk J.A., Doornaert P., Verdonck-de Leeuw I.M., Leemans C.R., Aaronson N.K., Slotman B.J. (2008). Impact of Late Treatment-Related Toxicity on Quality of Life among Patients with Head and Neck Cancer Treated with Radiotherapy. J. Clin. Oncol..

[97] Sciubba J.J., Goldenberg D. (2006). Oral Complications of Radiotherapy. Lancet Oncol..

[98] Little M., Schipper M., Feng F.Y., Vineberg K., Cornwall C., Murdoch-Kinch C.-A., Eisbruch A. (2012). Reducing Xerostomia After Chemo-IMRT for Head-and-Neck Cancer: Beyond Sparing the Parotid Glands. Int. J. Radiat. Oncol. Biol. Phys..

[99] Eisbruch A., Kim H.M., Terrell J.E., Marsh L.H., Dawson L.A., Ship J.A. (2001). Xerostomia and Its Predictors Following Parotid-Sparing Irradiation of Head-and-Neck Cancer. Int. J. Radiat. Oncol. Biol. Phys..

[100] Deasy J.O., Moiseenko V., Marks L., Chao K.S.C., Nam J., Eisbruch A. (2010). Radiotherapy Dose–Volume Effects on Salivary Gland Function. Int. J. Radiat. Oncol. Biol. Phys..

[101] Nutting C.M., Morden J.P., Harrington K.J., Urbano T.G., Bhide S.A., Clark C., Miles E.A., Miah A.B., Newbold K., Tanay M.A. (2011). Parotid-Sparing Intensity Modulated versus Conventional Radiotherapy in Head and Neck Cancer (PARSPORT): A Phase 3 Multicentre Randomised Controlled Trial. Lancet Oncol..

[102] Martinez J.R. (1987). Ion Transport and Water Movement. J. Dent. Res..

[103] Konings A.W.T., Coppes R.P., Vissink A. (2005). On the Mechanism of Salivary Gland Radiosensitivity. Int. J. Radiat. Oncol. Biol. Phys..

[104] Coppes R.P., Meter A., Latumalea S.P., Roffel A.F., Kampinga H.H. (2005). Defects in Muscarinic Receptor-Coupled Signal Transduction in Isolated Parotid Gland Cells after in Vivo Irradiation: Evidence for a Non-DNA Target of Radiation. Br. J. Cancer.

[105] Weng P.L., Aure M.H., Maruyama T., Ovitt C.E. (2018). Limited Regeneration of Adult Salivary Glands after Severe Injury Involves Cellular Plasticity. Cell Rep..

[106] Aure M.H., Konieczny S.F., Ovitt C.E. (2015). Salivary Gland Homeostasis Is Maintained through Acinar Cell Self-Duplication. Dev. Cell.

[107] Maimets M., Rocchi C., Bron R., Pringle S., Kuipers J., Giepmans B.N.G., Vries R.G.J., Clevers H., de Haan G., van Os R. (2016). Long-Term in Vitro Expansion of Salivary Gland Stem Cells Driven by Wnt Signals. Stem Cell Rep..

[108] Konings A.W.T., Cotteleer F., Faber H., van Luijk P., Meertens H., Coppes R.P. (2005). Volume Effects and Region-Dependent Radiosensitivity of the Parotid Gland. Int. J. Radiat. Oncol. Biol. Phys..

[109] Konings A.W.T., Faber H., Cotteleer F., Vissink A., Coppes R.P. (2006). Secondary Radiation Damage as the Main Cause for Unexpected Volume Effects: A Histopathologic Study of the Parotid Gland. Int. J. Radiat. Oncol. Biol. Phys..

[110] van Luijk P., Pringle S., Deasy J.O., Moiseenko V.V., Faber H., Hovan A., Baanstra M., van der Laan H.P., Kierkels R.G.J., van der Schaaf A. (2015). Sparing the Region of the Salivary Gland Containing Stem Cells Preserves Saliva Production after Radiotherapy for Head and Neck Cancer. Sci. Transl. Med..

[111] Buettner F., Miah A.B., Gulliford S.L., Hall E., Harrington K.J., Webb S., Partridge M., Nutting C.M. (2012). Novel Approaches to Improve the Therapeutic Index of Head and Neck Radiotherapy: An Analysis of Data from the PARSPORT Randomised Phase III Trial. Radiother. Oncol..

[112] Jiang W., Lakshminarayanan P., Hui X., Han P., Cheng Z., Bowers M., Shpitser I., Siddiqui S., Taylor R.H., Quon H. (2019). Machine Learning Methods Uncover Radiomorphologic Dose Patterns in Salivary Glands That Predict Xerostomia in Patients with Head and Neck Cancer. Adv. Radiat. Oncol..

[113] Miah A.B., Gulliford S.L., Morden J., Newbold K.L., Bhide S.A., Zaidi S.H., Hall E., Harrington K.J., Nutting C.M. (2016). Recovery of Salivary Function: Contralateral Parotid-Sparing Intensity-Modulated Radiotherapy versus Bilateral Superficial Lobe Parotid-Sparing Intensity-Modulated Radiotherapy. Clin. Oncol..

[114] Steenbakkers R.J.H.M. Parotid-Gland Stem-Cell Sparing Intensity-Modulated Radiotherapy (SCS-IMRT). https://clinicaltrials.gov/ct2/show/NCT01955239.

[115] Sari S.Y., Yilmaz M.T., Elmali A., Yedekci F.Y., Yuce D., Ozyigit G., Cengiz M., Yazici G. (2020). Parotid Gland Stem Cells: Mini yet Mighty. Head Neck.

[116] Robertson S.P., Quon H., Kiess A.P., Moore J.A., Yang W., Cheng Z., Afonso S., Allen M., Richardson M., Choflet A. (2015). A Data-Mining Framework for Large Scale Analysis of Dose-Outcome Relationships in a Database of Irradiated Head and Neck Cancer Patients. Med. Phys..

[117] van Luijk P., Faber H., Schippers J.M., Brandenburg S., Langendijk J.A., Meertens H., Coppes R.P. (2009). Bath and Shower Effects in the Rat Parotid Gland Explain Increased Relative Risk of Parotid Gland Dysfunction After Intensity-Modulated Radiotherapy. Int. J. Radiat. Oncol. Biol. Phys..

[118] Nagle P.W., Hosper N.A., Barazzuol L., Jellema A.L., Baanstra M., van Goethem M.J., Brandenburg S., Giesen U., Langendijk J.A., van Luijk P. (2018). Lack of DNA Damage Response at Low Radiation Doses in Adult Stem Cells Contributes to Organ Dysfunction. Clin. Cancer Res..

[119] Van Luijk P., Langendijk J.A., Coppes R.P. (2017). Understanding Mechanisms Yields Novel Approaches to Reduce Radiotherapy-Related Xerostomia. Ann. Transl. Med..

[120] Aps J.K.M., Martens L.C. (2005). Review: The Physiology of Saliva and Transfer of Drugs into Saliva. Forensic Sci. Int..

[121] Dawes C., Ong B.Y. (1973). Circadian Rhythms in the Concentrations of Protein and the Main Electrolytes in Human Unstimulated Parotid Saliva. Arch. Oral Biol..

[122] Dawes C. (1975). Circadian Rhythms in the Flow Rate and Composition of Unstimulated and Stimulated Human Submandibular Saliva. J. Physiol..

[123] Beetz I., Burlage F.R., Bijl H.P., Hoegen-Chouvalova O., Christianen M.E.M.C., Vissink A., van der Laan B.F.A.M., de Bock G.H., Langendijk J.A. (2010). The Groningen Radiotherapy-Induced Xerostomia Questionnaire: Development and Validation of a New Questionnaire. Radiother. Oncol..

[124] Dijkema T., Raaijmakers C.P.J., Braam P.M., Roesink J.M., Monninkhof E.M., Terhaard C.H.J. (2012). Xerostomia: A Day and Night Difference. Radiother. Oncol..

[125] Beetz I., Schilstra C., Visink A., van der Schaaf A., Bijl H.P., van der Laan B.F.A.M., Steenbakkers R.J.H.M., Langendijk J.A. (2013). Role of Minor Salivary Glands in Developing Patient-Rated Xerostomia and Sticky Saliva during Day and Night. Radiother. Oncol..

[126] Marks L.B., Bentzen S.M., Deasy J.O., Kong F.-M., Bradley J.D., Vogelius I.S., El Naqa I., Hubbs J.L., Lebesque J.V., Timmerman R.D. (2010). Radiation Dose Volume Effects in the Lung. Int. J. Radiat. Oncol. Biol. Phys..

[127] Graham M.V., Purdy J.A., Emami B., Harms W., Bosch W., Lockett M.A., Perez C.A. (1999). Clinical Dose-Volume Histogram Analysis for Pneumonitis after 3D Treatment for Non-Small Cell Lung Cancer (NSCLC). Int. J. Radiat. Oncol. Biol. Phys..

[128] Yorke E.D., Jackson A., Rosenzweig K.E., Merrick S.A., Gabrys D., Venkatraman E.S., Burman C.M., Leibel S.A., Ling C.C. (2002). Dose-Volume Factors Contributing to the Incidence of Radiation Pneumonitis in Non-Small-Cell Lung Cancer Patients Treated with Three-Dimensional Conformal Radiation Therapy. Int. J. Radiat. Oncol. Biol. Phys..

[129] Liao Z.X., Travis E.L., Tucker S.L. (1995). Damage and Morbidity from Pneumonitis after Irradiation of Partial Volumes of Mouse Lung. Int. J. Radiat. Oncol. Biol. Phys..

[130] Travis E.L., Liao Z.-X., Tucker S.L. (1997). Spatial Heterogeneity of the Volume Effect for Radiation Pneumonitis in Mouse Lung. Int. J. Radiat. Oncol. Biol. Phys..

[131] Khan M.A., Hill R.P., van Dyk J. (1998). Partial Volume Rat Lung Irradiation: An Evaluation of Early DNA Damage. Int. J. Radiat. Oncol. Biol. Phys..

[132] Khan M.A., van Dykc J., Yeunge I.W.T., Hilla R.P. (2003). Partial Volume Rat Lung Irradiation; Assessment of Early DNA Damage in Different Lung Regions and Effect of Radical Scavengers. Radiother. Oncol..

[133] Boersma L., Theuws J., Kwa S., Damen E., JV L. (1995). Regional Variation in Functional Subunit Density of the Lung: Regarding Liao et Al IJROBP 32(5):1359–1370; 1995. Int. J. Radiat. Oncol. Biol. Phys..

[134] Novakova-Jiresova A., van Luijk P., Van Goor H., Kampinga H.H., Coppes R.P. (2005). Pulmonary Radiation Injury: Identification of Risk Factors Associated with Regional Hypersensitivity. Cancer Res..

[135] Ghobadi G., Bartelds B., van der Veen S.J., Dickinson M.G., Brandenburg S., Berger R.M.F., Langendijk J.A., Coppes R.P., van Luijk P. (2012). Lung Irradiation Induces Pulmonary Vascular Remodelling Resembling Pulmonary Arterial Hypertension. Thorax.

[136] Coppes R.P., Muijs C.T., Faber H., Gross S., Schippers J.M., Brandenburg S., Langendijk J.A., van Luijk P. (2011). Volume-Dependent Expression of In-Field and Out-of-Field Effects in the Proton-Irradiated Rat Lung. Int. J. Radiat. Oncol. Biol. Phys..

[137] Kandathil A., Chamarthy M. (2018). Pulmonary Vascular Anatomy & Anatomical Variants. Cardiovasc. Diagn. Ther..

[138] Defraene G., van Elmpt W., Crijns W., de Ruysscher D. (2017). Regional Variability in Radiation-Induced Lung Damage Can Be Predicted by Baseline CT Numbers. Radiother. Oncol..

[139] Stam B., Kwint M., Guckenberger M., Mantel F., Hope A., Giuliani M., Werner-Wasik M., Grills I., Sonke J.-J., Belderbos J. (2019). Subgroup Survival Analysis in Stage I-II NSCLC Patients with a Central Tumor Partly Treated with Risk-Adapted SBRT. Int. J. Radiat. Oncol. Biol. Phys..

[140] Schlaak R.A., Senthilkumar G., Boerma M., Bergom C. (2020). Advances in Preclinical Research Models of Radiation-Induced Cardiac Toxicity. Cancers.

[141] Ghita M., Gill E.K., Walls G.M., Edgar K.S., McMahon S.J., Osorio E.V., Bergom C., Grieve D.J., Watson C.J., McWilliam A. (2020). Cardiac Sub-Volume Targeting Demonstrates Regional Radiosensitivity in the Mouse Heart. Radiother. Oncol..

[142] Stam B., Peulen H., Guckenberger M., Mantel F., Hope A., Werner-Wasik M., Belderbos J., Grills I., O’Connell N., Sonke J.J. (2017). Dose to Heart Substructures Is Associated with Non-Cancer Death after SBRT in Stage I–II NSCLC Patients. Radiother. Oncol..

[143] McWilliam A., Kennedy J., Hodgson C., Vasquez Osorio E., Faivre-Finn C., van Herk M. (2017). Radiation Dose to Heart Base Linked with Poorer Survival in Lung Cancer Patients. Eur. J. Cancer.

[144] McWilliam A., Khalifa J., Vasquez Osorio E., Banfill K., Abravan A., Faivre-Finn C., van Herk M. (2020). Novel Methodology to Investigate the Effect of Radiation Dose to Heart Substructures on Overall Survival. Int. J. Radiat. Oncol. Biol. Phys..

[145] Hotca A., Thor M., Deasy J.O., Rimner A. (2019). Dose to the Cardio-Pulmonary System and Treatment-Induced Electrocardiogram Abnormalities in Locally Advanced Non-Small Cell Lung Cancer. Clin. Transl. Radiat. Oncol..

[146] van Luijk P., Novakova-Jiresova A., Faber H., Schippers J.M., Kampinga H.H., Meertens H., Coppes R.P. (2005). Radiation Damage to the Heart Enhances Early Radiation-Induced Lung Function Loss. Cancer Res..

[147] Ghobadi G., van der Veen S., Bartelds B., de Boer R.A., Dickinson M.G., de Jong J.R., Faber H., Niemantsverdriet M., Brandenburg S., Berger R.M.F. (2012). Physiological Interaction of Heart and Lung in Thoracic Irradiation. Int. J. Radiat. Oncol. Biol. Phys..

[148] Tucker S.L., Liao Z., Dinh J., Bian S.X., Mohan R., Martel M.K., Grosshans D.R. (2014). Is There an Impact of Heart Exposure on the Incidence of Radiation Pneumonitis? Analysis of Data from a Large Clinical Cohort. Acta Oncol..

[149] Huang E.X., Hope A.J., Lindsay P.E., Trovo M., El Naqa I., Deasy J.O., Bradley J.D. (2011). Heart Irradiation as a Risk Factor for Radiation Pneumonitis. Acta Oncol..

[150] Teinturier C., Tournade M.-F., Caillat-Zucman S., Boitard C., Amoura Z., Bougneres P.-F., Timsit J. (1995). Diabetes Mellitus after Abdominal Radiation Therapy. Lancet.

[151] de Vathaire F., El-Fayech C., Ben Ayed F.F., Haddy N., Guibout C., Winter D., Thomas-Teinturier C., Veres C., Jackson A., Pacquement H. (2012). Radiation Dose to the Pancreas and Risk of Diabetes Mellitus in Childhood Cancer Survivors: A Retrospective Cohort Study. Lancet Oncol..

[152] Friedman D.N., Moskowitz C.S., Hilden P., Howell R.M., Weathers R.E., Smith S.A., Wolden S.L., Tonorezos E.S., Mostoufi-Moab S., Chow E.J. (2020). Radiation Dose and Volume to the Pancreas and Subsequent Risk of Diabetes Mellitus: A Report from the Childhood Cancer Survivor Study. JNCI J. Natl. Cancer Inst..

[153] van Nimwegen F.A., Schaapveld M., Janus C.P.M., Krol A.D.G., Raemaekers J.M.M., Kremer L.C.M., Stovall M., Aleman B.M.P., van Leeuwen F.E. (2014). Risk of Diabetes Mellitus in Long-Term Survivors of Hodgkin Lymphoma. J. Clin. Oncol..

[154] Lobo N., Kulkarni M., Hughes S., Nair R., Khan M.S., Thurairaja R. (2018). Urologic Complications Following Pelvic Radiotherapy. Urology.

[155] Zuppone S., Bresolin A., Spinelli A.E., Fallara G., Lucianò R., Scarfò F., Benigni F., Di Muzio N., Fiorino C., Briganti A. (2020). Pre-Clinical Research on Bladder Toxicity After Radiotherapy for Pelvic Cancers: State-of-the Art and Challenges. Front. Oncol..

[156] Mylona E., Ebert M., Kennedy A., Joseph D., Denham J., Steigler A., Supiot S., Acosta O., de Crevoisier R. (2020). Rectal and Urethro-Vesical Subregions for Toxicity Prediction After Prostate Cancer Radiation Therapy: Validation of Voxel-Based Models in an Independent Population. Int. J. Radiat. Oncol. Biol. Phys..

[157] Viana R., Batourina E., Huang H., Dressler G.R., Kobayashi A., Behringer R.R., Shapiro E., Hensle T., Lambert S., Mendelsohn C. (2007). The Development of the Bladder Trigone, the Center of the Anti-Reflux Mechanism. Development.

[158] Improta I., Palorini F., Cozzarini C., Rancati T., Avuzzi B., Franco P., Degli Esposti C., Del Mastro E., Girelli G., Iotti C. (2016). Bladder Spatial-Dose Descriptors Correlate with Acute Urinary Toxicity after Radiation Therapy for Prostate Cancer. Phys. Med..

[159] Heemsbergen W.D., Al-Mamgani A., Witte M.G., van Herk M., Pos F.J., Lebesque J.V. (2010). Urinary Obstruction in Prostate Cancer Patients from the Dutch Trial (68 Gy vs. 78 Gy): Relationships with Local Dose, Acute Effects, and Baseline Characteristics. Int. J. Radiat. Oncol. Biol. Phys..

[160] Ghadjar P., Zelefsky M.J., Spratt D.E., Munck af Rosenschöld P., Oh J.H., Hunt M., Kollmeier M., Happersett L., Yorke E., Deasy J.O. (2014). Impact of Dose to the Bladder Trigone on Long-Term Urinary Function After High-Dose Intensity Modulated Radiation Therapy for Localized Prostate Cancer. Int. J. Radiat. Oncol. Biol. Phys..

[161] Schaake W., van der Schaaf A., van Dijk L.V., van den Bergh A.C.M., Langendijk J.A. (2018). Development of a Prediction Model for Late Urinary Incontinence, Hematuria, Pain and Voiding Frequency among Irradiated Prostate Cancer Patients. PLoS ONE.

[162] Colaco R.J., Martin P., Kluger H.M., Yu J.B., Chiang V.L. (2016). Does Immunotherapy Increase the Rate of Radiation Necrosis after Radiosurgical Treatment of Brain Metastases?. J. Neurosurg..

[163] Tran T.T., Jilaveanu L.B., Omuro A., Chiang V.L., Huttner A., Kluger H.M. (2019). Complications Associated with Immunotherapy for Brain Metastases. Curr. Opin. Neurol..

[164] Koper P.C.M., Heemsbergen W.D., Hoogeman M.S., Jansen P.P., Hart G.A.M., Wijnmaalen A.J., van Os M., Boersma L.J., Lebesque J.V., Levendag P. (2004). Impact of Volume and Location of Irradiated Rectum Wall on Rectal Blood Loss after Radiotherapy of Prostate Cancer. Int. J. Radiat. Oncol. Biol. Phys..

[165] Maruyama C., Monroe M., Hunt J., Buchmann L., Baker O. (2019). Comparing Human and Mouse Salivary Glands: A Practice Guide for Salivary Researchers. Oral Dis..

[166] Jackson I.L., Vujaskovic Z., Down J.D. (2011). A Further Comparison of Pathologies after Thoracic Irradiation among Different Mouse Strains: Finding the Best Preclinical Model for Evaluating Therapies Directed Against Radiation-Induced Lung Damage. Radiat. Res..

[167] Groves A.M., Johnston C.J., Misra R.S., Williams J.P., Finkelstein J.N. (2015). Whole-Lung Irradiation Results in Pulmonary Macrophage Alterations That Are Subpopulation and Strain Specific. Radiat. Res..

[168] Widder J. Evaluation of Radiation Induced Pulmonary Hypertension Using MRI in Stage III NSCLC Patients Treated with Chemoradiotherapy. A Pilot Study (MRI-HART). https://clinicaltrials.gov/ct2/show/NCT02377934.

[169] Qiu Y., Guo Z., Han L., Yang Y., Li J., Liu S., Lv X. (2018). Network-Level Dysconnectivity in Patients with Nasopharyngeal Carcinoma (NPC) Early Post-Radiotherapy: Longitudinal Resting State FMRI Study. Brain Imaging Behav..

[170] Suckert T., Müller J., Beyreuther E., Azadegan B., Brüggemann A., Bütof R., Dietrich A., Gotz M., Haase R., Schürer M. (2020). High-Precision Image-Guided Proton Irradiation of Mouse Brain Sub-Volumes. Radiother. Oncol..

[171] Andrianova N.V., Buyan M.I., Zorova L.D., Pevzner I.B., Popkov V.A., Babenko V.A., Silachev D.N., Plotnikov E.Y., Zorov D.B. (2019). Kidney Cells Regeneration: Dedifferentiation of Tubular Epithelium, Resident Stem Cells and Possible Niches for Renal Progenitors. Int. J. Mol. Sci..

[172] Dawson L.A., Kavanagh B.D., Paulino A.C., Das S.K., Miften M., Li X.A., Pan C., Ten Haken R.K., Schultheiss T.E. (2010). Radiation-Associated Kidney Injury. Int. J. Radiat. Oncol. Biol. Phys..

[173] Lopez-Gaitan J., Ebert M.A., Robins P., Boucek J., Leong T., Willis D., Bydder S., Podias P., Waters G., O’Mara B. (2013). Radiotherapy of Abdomen with Precise Renal Assessment with SPECT/CT Imaging (RAPRASI): Design and Methodology of a Prospective Trial to Improve the Understanding of Kidney Radiation Dose Response. BMC Cancer.

[174] Hawkins M.M., Smith R.A. (1989). Pregnancy Outcomes in Childhood Cancer Survivors: Probable Effects of Abdominal Irradiation. Int. J. Cancer.

[175] Signorello L.B., Mulvihill J.J., Green D.M., Munro H.M., Stovall M., Weathers R.E., Mertens A.C., Whitton J.A., Robison L.L., Boice J.D. (2010). Stillbirth and Neonatal Death in Relation to Radiation Exposure before Conception: A Retrospective Cohort Study. Lancet.

[176] Sudour H., Chastagner P., Claude L., Desandes E., Klein M., Carrie C., Bernier V. (2010). Fertility and Pregnancy Outcome After Abdominal Irradiation That Included or Excluded the Pelvis in Childhood Tumor Survivors. Int. J. Radiat. Oncol. Biol. Phys..

[177] Jadon R., Higgins E., Hanna L., Evans M., Coles B., Staffurth J. (2019). A Systematic Review of Dose-Volume Predictors and Constraints for Late Bowel Toxicity Following Pelvic Radiotherapy. Radiat. Oncol..

[178] Maeda Y., Høyer M., Lundby L., Norton C. (2011). Faecal Incontinence Following Radiotherapy for Prostate Cancer: A Systematic Review. Radiother. Oncol..

[179] Chan C., Facer P., Davis J., Smith G., Egerton J., Bountra C., Williams N., Anand P. (2003). Sensory Fibres Expressing Capsaicin Receptor TRPV1 in Patients with Rectal Hypersensitivity and Faecal Urgency. Lancet.

